# The molecular clock gene cryptochrome 1 (*CRY1*) and its role in cluster headache

**DOI:** 10.1177/03331024211024165

**Published:** 2021-07-13

**Authors:** Carmen Fourier, Caroline Ran, Christina Sjöstrand, Elisabet Waldenlind, Anna Steinberg, Andrea Carmine Belin

**Affiliations:** 1 Department of Neuroscience, 27106 Karolinska Institutet, Karolinska Institutet, Stockholm, Sweden; 2 Department of Clinical Neuroscience, 27106 Karolinska Institutet, Karolinska Institutet, Stockholm, Sweden; 3 Department of Neurology, 59562 Karolinska University Hospital, Karolinska University Hospital, Stockholm, Sweden

**Keywords:** circadian rhythm, single nucleotide polymorphism, mRNA expression, case-control study, genetic association study, cluster headache genetics

## Abstract

**Background:**

Cluster headache is a severe primary headache disorder commonly featuring a strikingly distinct circadian attack pattern. Therefore, the circadian system has been suggested to play a crucial role in the pathophysiology of cluster headache. Cryptochromes are key components of the molecular clock generating circadian rhythms and have previously been shown to be associated with several psychiatric disorders, including seasonal affective disorder, bipolar disorder, and depression.

**Methods:**

In this case-control study, we investigated the role of cryptochrome (*CRY*) genes in cluster headache by screening 628 cluster headache patients and 681 controls from Sweden for four known genetic variants in the *CRY1* (rs2287161 and rs8192440) and *CRY2* (rs10838524 and rs1554338) genes. In addition, we analyzed *CRY1* gene expression in primary fibroblast cell lines from eleven patients and ten controls.

**Results:**

The exonic *CRY1* variant rs8192440 was associated with cluster headache on allelic level (*p*=0.02) and this association was even more pronounced in a subgroup of patients with reported diurnal rhythmicity of attacks (*p*=0.002). We found a small significant difference in *CRY1* gene expression between cluster headache patients and control individuals (*p*=0.04), but we could not identify an effect of the associated variant rs8192440 on *CRY1* expression.

**Conclusions:**

We discovered a disease-associated variant in the *CRY1* gene and slightly increased *CRY1* gene expression in tissue from cluster headache patients, strengthening the hypothesis of circadian dysregulation in cluster headache. How this gene variant may contribute to the pathophysiology of the disease remains subject to further studies.

## Introduction

A striking feature of cluster headache (CH) is the circadian and circannual attack pattern by which the attacks repeatedly occur. Diurnal rhythmicity of headache attacks occurs in 67–82% of all CH patients, therefore it is hypothesized that the circadian system is dysregulated in CH (1–3).

The hypothalamus may play a critical role in the pathophysiology of CH which is underlined by different studies demonstrating its activation during CH attacks but also outside of bouts (4,5). The central pacemaker, the suprachiasmatic nucleus (SCN), is located in the anterior hypothalamus and synchronizes the cellular clocks in the periphery generating circadian rhythms. The cellular clock in mammals is driven by feedback loops of several intracellular core clock components: circadian locomotor output cycles kaput (CLOCK), brain and muscle ARNT-like 1 (BMAL1), period circadian regulator 1, 2 and 3 (PERs), and cryptochrome 1 and 2 (CRYs). The heterodimers CLOCK/BMAL1 and PERs/CRYs activate and inhibit each other producing a cell-autonomous oscillation of approximately 24 hours (6,7).

One genetic study has found an association between CH and the *CLOCK* gene, but there are conflicting results from three smaller case-control studies (<107 CH patients and <210 controls) (8–12). A *PER3* gene variant has been screened in a Norwegian CH cohort, but no association was found (13). The *NR1D1* gene, encoding for an important transcriptional repressor of *BMAL1*, was found to have significantly decreased expression in lymphoblasts from CH patients compared to controls (14). In line with these results, the present study investigated the involvement of the *CRY* genes in CH. The cryptochrome family consists of two proteins encoded by the genes *CRY1* (on chromosome 12) and *CRY2* (on chromosome 11). Interestingly, a study has demonstrated that CRY1 is a much more potent repressor of CLOCK/BMAL1-mediated transcription than CRY2 (15). The two *CRY* genes have previously been studied in a number of different neurological and psychiatric disorders; *CRY1* was reported to be associated with depression, familial delayed sleep phase disorder, and schizophrenia (16–18). Other studies have found a link between *CRY2* and depression, seasonal affective disorder, dysthymia, and bipolar disorder (19–22).

The objective of this study was to investigate the role of cryptochrome (*CRY*) genes in cluster headache. Four single nucleotide polymorphisms (SNPs) have been chosen for screening in our Swedish CH case-control material due to evidence of association with neurological and psychiatric disorders in previous studies and their potential effect on gene expression and/or function; rs2287161 (downstream of *CRY1*), rs8192440 (in exon 5 in *CRY1* leading to a silent mutation), rs10838524 (in intron 1 in *CRY2*), and rs1554338 (downstream of *CRY2*) (16,17,19,21). The aim of this study was to investigate if genetic variants in two genes involved in regulating circadian rhythm constitute risk factors for CH. Genetic risk factors will be identified by examining the frequencies of the different *CRY* variants in our large CH cohort and by analyzing *CRY* gene expression in biological tissue from CH patients and controls.

## Materials and methods

### Study population and material

Patients were recruited from across Sweden between 2014˗2017 to establish a Swedish CH biobank containing biological material (DNA samples and primary fibroblast cell lines) and questionnaire data (including clinical and lifestyle information), as described previously (table 1) (1). The majority of CH patients have reported a Swedish background (>90%). CH patients were asked if their attacks appear at certain time points divided into two-hour intervals or only at random time points and accordingly divided into two subgroups; 1) with diurnal rhythmicity and 2) without diurnal rhythmicity, respectively. Of these, 628 patients as well as 681 control individuals were investigated for genetic variations in the *CRY* genes. Of the 681 controls, 665 were anonymous blood donors, who represent a healthy Swedish population, and 16 were neurologically healthy control individuals recruited at Karolinska University Hospital in Stockholm, Sweden. The study material was obtained after approval of the Swedish Ethical Review Authority in Stockholm, Sweden (diary number 2014/656-31/4) and informed consent from all study participants. Patients were diagnosed with CH according to the International Classification of Headache Disorders (ICHD-III beta) criteria (23). DNA was purified from whole blood samples using the Gentra Puregene Blood Kit (QIAGEN, Hilden, Germany) according to manufacturer’s instructions, and DNA concentration was measured using the NanoDrop® ND-1000 Spectrophotometer (NanoDrop Technologies Inc., Wilmington, USA).

**Table 1.** Demographic characterization of cluster headache patients and controls.

	CH Patients	Controls
No. of individuals	628	681
Age (years)	52 ± 15 (17–92)	n/a
Age at onset (years)	32 ± 14 (7–70)	n/a
Male % (n)	68.3 (429)	54.8 (373)
Family history† % (n)	11.7 (69)	n/a
Diurnal rhythmicity† % (n)	65.5 (386)	n/a

Age presented as mean ± SD (range). †Based on 589 individuals for whom detailed information was available. Family history includes first-, second-, and third-degree relatives with a CH diagnosis. CH, cluster headache; n/a, not available/applicable.

### Genotyping

Genotyping of two *CRY1* (rs2287161 and rs8192440) and two *CRY2* (rs10838524 and rs1554338) SNPs was performed using quantitative Real-Time PCR (qPCR). We used pre-designed TaqMan® SNP genotyping assays (C__11305584_1_, C__30476859_10, C____497931_10, C___8894868_10), TaqMan® Genotyping Master Mix, and 5 ng DNA. The ABI 7500 Fast Real-Time PCR instrument (Applied Biosystems, Foster City, CA, USA) was programmed according to the manufacturer’s recommendations with increased cycle number. Samples with missing genotype data were excluded from the analysis of the individual SNP as well as the haplotype analysis. Genotyping data were analyzed using the software supplied with the instrument, SDS v.2.0.4. For power analysis, the power and sample size calculation software PS v3.1.6 was used (24). With our sample size and a minor allele frequency between 0.06–0.46 in individuals with European descent (*https://www.ncbi.nlm.nih.gov/snp/docs/gsr/alfa/*), we have 80% power to detect true odds ratios (OR) for CH below 0.43–0.73 or above 1.37–1.82, depending on the SNP. All SNPs were in Hardy-Weinberg equilibrium (HWE) for both cases and controls, except for rs10838524 where controls deviated from HWE. Genotype association was evaluated with chi-square (χ^2^) test and allele association was analyzed with Fisher’s exact test using GraphPad Prism version 8.0.1 for Windows (GraphPad Software, San Diego, CA, USA, *www.graphpad.com*). All tests were two-tailed with a significance level <0.05 and were run with Bonferroni correction for multiple testing, *p_corrected_* (*p_c_*), when applicable. For a stratified analysis, genotype and allele frequencies for all four SNPs were compared between a patient subgroup who reported a diurnal pattern for their CH attacks (n=386) and controls. Effect sizes are presented as odds ratio (OR) with 95% confidence interval (CI) for the minor allele. Haplotype analysis and permutation of association results to correct for multiple testing bias (10,000 permutations) were performed using HaploView v4.2 (25).

### RNA folding prediction

To evaluate a possible effect of rs8192440 on *CRY1* mRNA secondary structure, RNA folding was predicted using the mfold web server (26). A partial *CRY1* mRNA sequence of 141 nucleotides including flanking sequences (70 kb) on either side of the mutation was analyzed and compared to the wild type sequence.

### Gene expression

For gene expression experiments, skin biopsies from eleven CH patients and ten healthy control individuals were obtained. From the biopsies, primary fibroblast cell cultures were established according to protocol (27). Cells were given a high-serum shock to synchronize their circadian clocks, since *CRY1* displays a rhythmically oscillating expression throughout the day (18). Fibroblasts were harvested at 12 hours after serum shock treatment (*zeitgeber* time +12h), a time point when CRY1 gene expression was easily detectable and stable in controls, and frozen at –80°C. RNA and cDNA were prepared using standard procedures. The qPCR was performed using 200 ng cDNA per well, iTaq™ Universal SYBR® Green Supermix, and previously published *CRY1* primers (F: 5′-ATGATCCCTGGAATGCACCA-3′; R: 5′-CGATATTCAAACGGCTTGCCT -3′) (28). As endogenous controls, the reference genes *TBP* (TATA-binding protein; F: 5′-AGGCAACACAGGGAACCTC-3′; R: 5′-TTGCAGCTGCGGTACAATCC-3′) and *IPO8* (importin-8; F: 5′-ATTGGAAGAAACCGCGCTTG-3′; R: 5′-TGTGTACACCTCCTGCAGTG-3′) were chosen due to previously shown stable expression in CH (29). A pool of the control samples comprised the reference sample. The Bio-Rad CFX384 Touch Real-Time PCR Detection System (Bio-Rad Laboratories, Inc., Hercules, CA, USA) was programmed as follows: polymerase activation for 3 minutes at 95°C, denaturation for 3 sec at 95°C and annealing/extension for 30 sec at 60°C for 40 cycles followed by a melting curve. To detect outliers, Grubb’s test was applied to the log_2_-transformed values in the patient and control group separately, none were detected. Gene expression data were analyzed using Bio-Rad CFX Manager v3.1, and statistical analysis was performed in GraphPad Prism using Mann-Whitney *U*-test to compare cases and controls, and Kruskal-Wallis test to compare the three different rs8192440 genotypes. Both tests were two-tailed with a significance level <0.05. For one control individual, genotype data was not available. Data presented as mean log_2_-transformed expression ± standard deviation (SD). Expression quantitative trait loci (eQTL) data was retrieved from the publicly accessible Genotype-Tissue Expression (GTEx) project website (*www.gtexportal.org*).

## Results

### Study participants

At the time of genotyping, a total of 1065 validated CH patients had been contacted for participation and blood from 628 participants had been obtained. Among the remaining patients, 13 were deceased before follow-up, 31 did not wish to participate, and 393 had not replied yet at the time of this study.

We have genotyped 628 CH patients and 681 control individuals for four different genetic variants in the *CRY* genes, two SNPs in *CRY1* and two in *CRY2*. Genotyping call rate was 98.9%. Demographic and clinical information for the study participants has been summarized in Table 1. The patient group contained predominantly male participants (68.3% men), while the control group was more balanced (54.8% men). Of our CH patients, 65.5% of those who additionally completed a questionnaire reported that during a bout their CH attacks recurred periodically at specific times of the day.

### Genotypic and allelic analysis of CRY1 and CRY2 variants

Genotyping results are summarized in Table 2. For the *CRY1* variant rs8192440, the major allele G was more common in patients than in control individuals, even after Bonferroni correction (0.80 [0.68–0.94], *p_c_*=0.023) with no difference between the sexes (data available upon request). This association was even more pronounced for both genotype (*p_c_*=0.004) and allele frequency (0.72 [0.60–0.87], *p_c_*=0.002) when comparing controls to only a subgroup of 385 CH patients whose attacks occurred with diurnal rhythmicity (Table 3). We could not identify a significant difference in neither genotype nor allele frequencies between patients and controls for rs2287161 or the *CRY2* variants rs10838524 and rs1554338 in relation to diurnal rhythmicity (data available upon request). A multi-marker analysis for the two *CRY1* variants revealed a haplotype including the minor allele C for rs2287161 and the major allele G for rs8192440 to be associated with CH which holds even after permutation testing (*p_c_*=0.024, Table 4).

**Table 2.** Genotype and allele frequencies for two *CRY1* and two *CRY2* SNPs in CH patients and controls.

Gene	SNP	Genotype/Allele	Control % (n)	CH % (n)	χ^2^ (df)	OR (95% CI)	*p*-value	*p_c_*-value
CRY1	rs2287161	GG	29.7 (202)	27.2 (171)	0.63 (2)		0.73	>1.0
		GC	50.5 (344)	50.6 (318)				
		CC	19.8 (135)	20.4 (128)				
		G	54.9 (748)	53.5 (660)		1.06 (0.91–1.24)	0.48	>1.0
		C	45.1 (614)	46.5 (574)				
	rs8192440	GG	32.5 (221)	39.3 (247)	8.06 (2)		0.018	0.07
		GA	50.4 (343)	47.8 (300)				
		AA	16.6 (113)	12.7 (80)				
		G	58.0 (785)	63.3 (794)		0.80 (0.68–0.94)	0.006	0.023*
		A	42.0 (569)	36.7 (460)				
CRY2	rs10838524	GG	32.3 (220)	31.8 (200)	0.79 (2)		0.67	>1.0
		GA	42.9 (292)	46.8 (294)				
		AA	20.7 (141)	20.5 (129)				
		G	56.0 (732)	55.7 (694)		1.01 (0.87–1.18)	0.87	>1.0
		A	44.0 (574)	44.3 (552)				
	rs1554338	AA	87.7 (597)	86.5 (543)	0.34 (2)		0.84	>1.0
		AG	11.2 (76)	12.1 (76)				
		GG	0.7 (5)	0.6 (4)				
		A	93.7 (1270)	93.3 (1162)		1.07 (0.78–1.46)	0.69	>1.0
		G	6.3 (86)	6.7 (84)				

SNP, single nucleotide polymorphism; CH, cluster headache; χ^2^, chi-squared; df, degrees of freedom; OR, odds ratio; CI, confidence interval; *p_c_*, corrected p-value after Bonferroni correction for multiple testing; *p_c_-value < 0.05 was considered signifcant.

**Table 3.** Genotype and allele frequencies for *CRY1* SNP rs8192440 in CH patients with diurnal rhythmicity and controls.

Gene	SNP name	Genotype/Allele	Control % (n)	CH_diurnal_ % (n)	χ^2^ (df)	OR (95% CI)	*p*-value	*p_c_*-value
CRY1	rs8192440	GG	32.5 (221)	43.8 (169)	13.7 (2)		0.001	0.004*
		GA	50.4 (343)	43.3 (167)				
		AA	16.6 (113)	12.7 (49)				
		G	58.0 (785)	65.6 (505)		0.72 (0.60–0.87)	<0.001	0.002*
		A	42.0 (569)	34.4 (265)				

SNP, single nucleotide polymorphism; CH_diurnal_, cluster headache patients reporting attacks to occur with diurnal rhythmicity; χ^2^, chi-square; df, degrees of freedom; OR, odds ratio; CI, confidence interval; *p_c_*, corrected p-value after Bonferroni multiple testing; *p_c_-value < 0.05 was considered significant.

**Table 4.** Haplotype analysis for two *CRY1* SNPs in CH patients and controls.

Haplotype	Controls % (n)	CH Patients % (n)	χ^2^ (df=2)	*p*-value	*p_c_*-value
G-G‡	34.8 (471)	36.4 (444)	0.67	0.414	0.757
C-G	23.2 (314)	27.3 (333)	5.93	0.015	0.024*
C-A	22.0 (298)	19.3 (236)	2.91	0.088	0.184
G-A	20.0 (271)	17.0 (207)	3.77	0.052	0.107

‡Reference haplotype (G-G) corresponds to major allele of each *CRY1* single nucleotide polymorphism (SNP) where rs2287161 is in position 1, and rs8192440 in position 2. CH, cluster headache; χ^2^, chi-square; df, degrees of freedom; *p_c_*, corrected p-value after permutation testing (10,000 permutations); *p_c_-value < 0.05 was considered significant.

### Prediction of CRY1 partial RNA secondary structure

**Figure 1.**
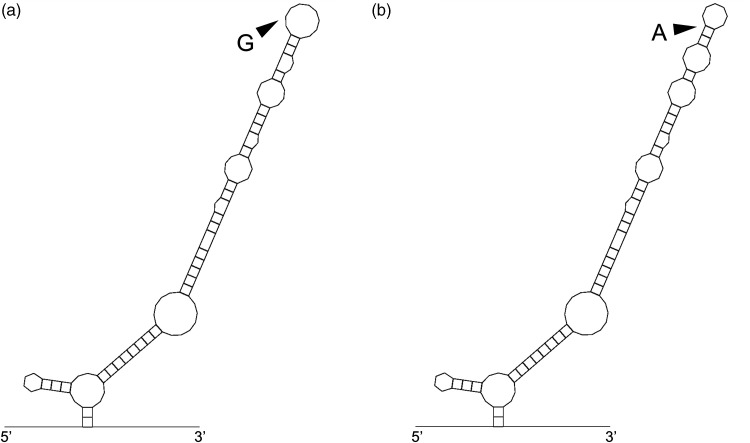
Effect of rs8192440 on *CRY1* mRNA secondary structure. a) The effect of the rs8192440 (G>A) mutation on the mRNA structure of *CRY1* was estimated to be a 2.9% difference in secondary structure and energy for RNA folding (ΔG) between mRNA sequences with (a) the major allele G (ΔG = –33.8 kcal/mol) or b) minor allele A (ΔG = –34.8 kcal/mol).

In order to evaluate whether the associated synonymous variant rs8192440 may influence the secondary structure of *CRY1* mRNA, we performed an RNA folding prediction on a partial *CRY1* mRNA sequence flanking the two possible alleles for rs8192440 (Figure 1). For the RNA sequence including the major allele G, the free energy for RNA folding (ΔG) is –33.8 kcal/mol, and for the sequence with the minor allele A, ΔG = –34.8 kcal/mol, resulting in a 2.9% difference in RNA folding energy.

### CRY1 gene expression analysis

**Figure 2.**
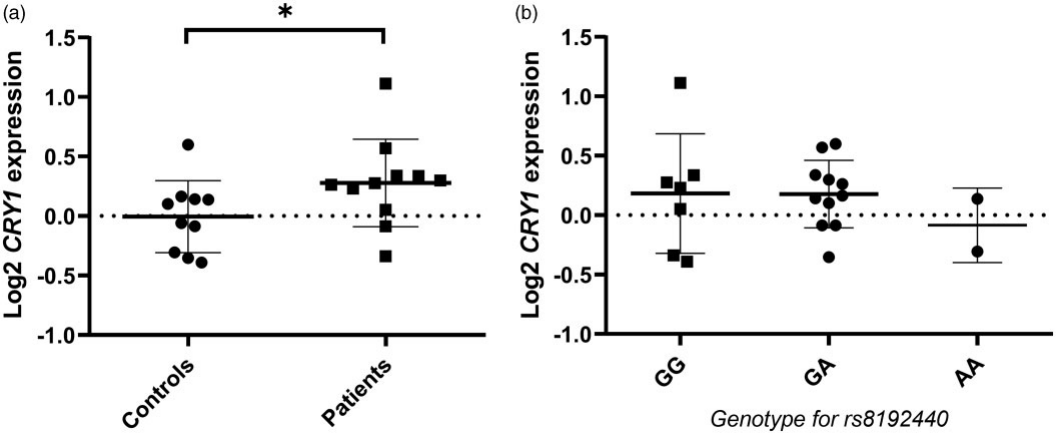
*CRY1* mRNA expression in correlation to disease status or genotype. a) Quantification of log2-transformed *CRY1* mRNA levels in human fibroblasts from controls (n = 10) and cluster headache patients (n = 11), normalized to the housekeeping genes *TBP* and *IPO8* as well as a control reference sample, and compared using Mann-Whitney U-test (p = 0.04). *p-value < 0.05. b) *CRY1* mRNA expression levels in the same individuals grouped by the three different rs8192440 genotypes GG (n = 7), GA (n = 11), and AA (n = 2), compared using Kruskal-Wallis test (p = 0.60). Data presented as mean ± standard deviation in both graphs.

To investigate possible gene expression differences between CH patients and controls, we analyzed *CRY1* mRNA expression in synchronized fibroblast cell lines from eleven CH patients and ten control individuals at one time point (ZT+12h). We found a small but significant difference between controls and CH patients in relative *CRY1* gene expression (Figure 2A), with slightly higher relative *CRY1* expression in patients (0.278 ± 0.368 vs. ˗0.005 ± 0.302, *p*=0.04). Because of the association for CH with the *CRY1* SNP rs8192440, we grouped the samples by rs8192440 genotype, independent from disease phenotype (Figure 2B), but could not see a correlation between rs8192440 genotype and relative *CRY1* gene expression (GG/GA/AA: 0.182 ± 0.503 vs. 0.177 ± 0.284 vs, ˗0.085 ± 0.313, *p*=0.60). Hence, we could not confirm publicly available eQTL data showing a significant effect of rs8192440 on *CRY1* mRNA expression observed in human muscle˗skeletal tissue (*p*=5.1 × 10^−10^) and artery˗tibial tissue *(p*=0.00016).

## Discussion

The variant rs8192440 in the *CRY1* gene is associated with CH in our Swedish case-control material where the major allele G was more common in patients than control individuals. The association was even more distinct for a subgroup of patients with attacks occurring with diurnal rhythmicity. In addition, a *CRY1* haplotype including the rs8192440 major allele G was more common in cases compared to controls, but it was not stronger than the association of the rs8192440 polymorphism alone. Taken together, our genotyping results suggest that the major allele G of rs8192440 increases susceptibility for CH. This variant leads to a synonymous amino acid change in the CRY1 protein and may not directly affect protein structure. Nevertheless, synonymous mutations can for example result in reduced protein levels due to less stable mRNA, a different protein conformation by altering ribosomal pause sites on the mRNA, or even a non-functional protein caused by incorrect splicing of mRNA (30). By using RNA folding prediction algorithms, we found that the *CRY1* mRNA structure around this gene variant is slightly altered by the minor rs8192440 allele which, as a consequence, may affect its stability. However, we conclude that the difference in folding energy that we detected may not be sufficient to produce a significant change in RNA stability.

Furthermore, we investigated *CRY1* gene expression at one time point (ZT+12h) and detected higher relative expression levels in tissue from CH patients compared to controls. We could not confirm an effect of the rs8192440 variant on general *CRY1* mRNA expression, although publicly available eQTL data reports an increase in *CRY1* mRNA expression for each addition of a G allele. For our analysis, the number of samples in each group may simply have been too small in order to detect a significant correlation. Additionally, our results have been observed in skin tissue while significant eQTL data was obtained for muscle as well as arterial tissue. Nevertheless, our results on increased *CRY1* expression in CH patients agrees with eQTL data since CH patients have a higher frequency of the G allele. Therefore, we hypothesize that the major allele G of the genetic variant rs8192440 which is more common in CH leads to increased *CRY1* gene expression. High gene expression could result in increased protein levels, but gene and protein expression do not always correlate (31).

Changes in CRY1 protein expression levels have been implicated in mechanisms related to CH pathology such as changed cytokine levels (32). One study showed that CRY1 could regulate levels of proinflammatory cytokines in mice (33). As mentioned previously, the synonymous variant rs8192440 could also alter the CRY1 protein conformation. A recent study has illustrated that remodeling of a dynamic serine-rich loop in the CRY proteins can alter the affinity to CLOCK/BMAL1 (34). This is one example of how crucial a specific conformation is for the affinity of such a highly interacting protein like CRY1.

The strengths of this study are the large sample size and the well-characterized and validated CH cohort. However, the gene expression studies were made using a small subset of our material, eleven CH patients and ten controls. One potential limitation is the control cohort where a large majority consists of anonymous blood donors with only information on sex. Despite the small possibility that CH occurs in the control population, we consider this an excellent control group representing a healthy Swedish population between the ages of 18˗60 years (*https://geblod.nu/english/*). The skewed sex proportions in the patient group compared to the control group may introduce a potential source of bias. However, we have taken this into account in our analysis and can exclude that the associations found are driven by sex. Our power analysis indicates that this study may be slightly underpowered in order to detect the true effect size for rs8192440. For uncommon diseases, such as CH, it is challenging to conduct large genetic studies with sufficient power. In addition, CH is most likely a complex genetic disorder and therefore not caused by a single locus with large effect. In addition, we have only screened four genetic SNPs in *CRY1* and *CRY2* in our material and it is important to note that other *CRY* SNPs may have an effect on CH. Nevertheless, we believe that our results may contribute to a better understanding of CH pathophysiology.

By which mechanisms rs8192440 may affect the *CRY1* gene remains to be determined. Although more research is needed to consolidate our findings, for example an analysis of *CRY1* gene expression at different time points and CRY1 protein levels in CH patients, this study points to a role of the clock gene *CRY1* in CH pathophysiology and supports the hypothesis that the molecular clock is perturbed in CH.

## Key Findings

- A genetic variant in the cryptochrome 1 (*CRY1*) gene increases susceptibility for cluster headache in Sweden.
- The association between this *CRY1* gene variant and cluster headache is even more distinct in a patient subgroup reporting diurnal rhythmicity of attacks.
- *CRY1* gene expression is slightly increased in cluster headache patients compared to controls.
